# A High Preoperative Blood Urea Nitrogen to Serum Albumin Ratio Does Not Predict Worse Outcomes Following the Robotic-Assisted Pulmonary Lobectomy for Lung Cancer

**DOI:** 10.7759/cureus.50468

**Published:** 2023-12-13

**Authors:** Allison O Dumitriu Carcoana, Kristie M Labib, Cole R Fiedler, Jenna C Marek, Lauren C Ladehoff, William J West, Jose A Malavet, William N Doyle, Carla C Moodie, Joseph R Garrett, Jenna R Tew, Jobelle Joyce Anne R Baldonado, Jacques P Fontaine, Eric Toloza

**Affiliations:** 1 Medical Education, University of South Florida Health Morsani College of Medicine, Tampa, USA; 2 Thoracic Oncology, Moffitt Cancer Center, Tampa, USA; 3 Surgery and Oncologic Sciences, University of South Florida Health Morsani College of Medicine, Tampa, USA

**Keywords:** outcomes, pulmonary lobectomy, robotic surgery, lung cancer, serum albumin, blood urea nitrogen

## Abstract

Background: The blood urea nitrogen to serum albumin ratio (BAR) is an emerging prognostic parameter of interest. The utility of BAR as a prognostic factor has not been analyzed in lung cancer patients undergoing pulmonary lobectomy. We evaluated the ability of High BAR to predict worse outcomes after robotic-assisted pulmonary lobectomy (RAPL) for lung cancer.

Methods: We retrospectively analyzed 400 patients who underwent RAPL from September 2010 to March 2022 by one surgeon. Patients were stratified by Low BAR (<6.25 mg/g) and High BAR (≥6.25 mg/g). Patients’ demographics, tumor characteristics, comorbidities, surgical complications, outcomes, and survival were collected and compared by High and Low BAR groups. The primary outcome of interest was 30-day mortality.

Results: Receiver operator curves (ROC) confirmed that 6.25 was an optimal threshold for estimating mortality based on Low and High BAR. There were no differences in surgical complications or outcomes between the Low and High BAR groups. The ability of BAR to predict 30-day mortality was evaluated with the area under the curve (AUC) analysis, which showed that higher BAR could not predict mortality (AUC=0.655; 95% CI, 0.435-0.875; *p*=0.166). Similarly, survival analysis revealed no difference in five-year overall survival between the Low and High BAR groups (*p*=0.079).

Conclusion: High BAR did not predict worse outcomes after RAPL for lung cancer in our study. Further studies are needed to better determine the prognostic ability of BAR in lower-risk populations.

## Introduction

Cancer is the second leading cause of death in the United States [1]. Lung cancer is the leading cause of cancer death in the United States and worldwide [2]. Non-small cell lung cancer (NSCLC) is the most common type of lung cancer and is ideally treated with radical resection at an early stage [3]. Preoperative serum markers, including blood urea nitrogen (BUN) and albumin levels, have been studied as prognostic factors for lung cancer surgery [4-16].

A normal serum BUN level is in the range of 7-20 mg/dL for children, 6-21 mg/dL for adult women, and 8-24 mg/dL for adult men [17]. Elevated BUN levels reflect the function of many body systems and are associated with poor kidney function, dehydration, acute hemodynamic alterations, and acute neurohumoral alterations [7,18]. Therefore, high BUN has been correlated with increased mortality in many patient populations, including those with heart failure, acute myocardial infarction, pneumonia, exacerbation of chronic obstructive pulmonary disease, and after cranial tumor resection and surgical resection for pancreatic adenocarcinoma [19-23]. Often, elevated BUN is associated with elevations in creatinine, but Beier et al. reported that high BUN levels were correlated with increased short-term and long-term mortality after ICU admission, independent of creatinine levels [24]. A normal albumin level ranges from 3.5 to 5.5 g/dL [25]. Albumin is the most abundant plasma protein, involved in molecular transport, the regulation of colloid osmotic pressure, and pH buffering [26]. Low albumin levels were correlated with increased short- and long-term mortality after surgery and worse prognosis and survival in cancer patients [27-30]. In patients with nonmetastatic NSCLC, hypoalbuminemia has been identified as an independent predictor of worse five-year overall survival [31].

Recent studies have shown that an elevated BUN to serum albumin ratio (BAR) is predictive of worse post-surgical outcomes, ICU admission, and increased in-hospital and 30-day mortality in patients with several different illnesses [4-16]. A BAR greater than 6.25 mg/g has been correlated with an increased risk of in-hospital and in older emergency department patients. In that same study, elevated BAR was a more powerful independent predictor of in-hospital mortality than the individual BUN or albumin levels, as well as creatinine or estimated glomerular filtration rate (eGFR) [14]. Another study also reported that High BAR was a stronger predictor of in-hospital mortality than either BUN or serum albumin in ICU patients with lung cancer [15]. Kos et al. [31] studied the prognostic value of BAR in 142 patients with NSCLC, 36.6% of whom had stage IV disease. Using a cutoff value of 0.8, they found that the median survival was significantly higher in the Low BAR group, but this significance disappeared after multivariate analysis [31]. However, the use of BAR as a prognostic tool has not been assessed in patients undergoing surgical resection for NSCLC. The purpose of this current study is to evaluate the ability of BAR ≥6.25 mg/g to predict worse perioperative outcomes after robotic-assisted pulmonary lobectomy (RAPL) for lung cancer.

## Materials and methods

We retrospectively reviewed consecutive patients who underwent RAPL between September 2010 and March 2022 by one surgeon at a single cancer center. Data were extracted from patients’ electronic medical records and entered into a database on our institution’s secure network. Our institution’s Institutional Review Board approved this retrospective study protocol and waived the requirement to obtain informed consent. The inclusion criteria for patients in this study were age ≥18 years and having undergone elective RAPL by a specified surgeon for clinically diagnosed stage I/II/III NSCLC, with or without neoadjuvant therapy. The surgical procedure for RAPL in patients with clinically suspected or diagnosed lung cancer was carried out with the da Vinci® S™, Si™, or Xi™ robotic surgical systems (Intuitive Surgical Corp., Sunnyvale, CA, USA) depending on the date of surgery, according to a procedure detailed in Deol et al. [32].

The independent variable was BAR (mg/g), stratified as either Low BAR (BAR <6.25) or High BAR (BAR ≥6.25). Individual BAR was calculated by dividing the most recent preoperative BUN level (mg/dL) by the most recent preoperative serum albumin level (g/dL). Investigators evaluating the use of BAR as a prognostic factor in different cohorts have used cutoff BAR values ranging from as low as 0.83 mg/g to as high as 7.93 mg/g [4,11]. In our cohort, 6.25 mg/g was the optimal cutoff for predicting 30-day mortality. Several demographic and clinical variables were collected and compared between the High BAR and Low BAR groups. The primary outcome of interest was 30-day mortality.

Data were collected on patients’ medical histories, tumor characteristics, intraoperative complications, and perioperative and postoperative outcomes. Patients who quit smoking within three months prior to surgery and those who were actively smoking at the time of surgery were categorized as current smokers. Patients who quit at least three months prior to surgery were categorized as former smokers. We calculated Charlson Comorbidity Index (CCI) scores for each patient using 17 weighted comorbid conditions as a summary comorbidity measure. The CCI score estimates the risk of 10-year mortality from comorbid diseases and has been validated as a better predictor of long-term survival than individual comorbid conditions in patients who undergo surgery for NSCLC [33]. Patient disposition at discharge was categorized as favorable if they were discharged home with self-care or with home health nursing and/or physical therapy and unfavorable if they were discharged to a long-term acute care facility, rehabilitation facility, hospice, or if the patient expired. The date of last follow-up was utilized in survival analysis and was defined as the date a patient was last seen in the clinic, last contacted, or their date of death. For survival analysis, Kaplan-Meier curves were generated to estimate five-year overall survival with 95% confidence intervals.

We reported the mean and standard error of the mean (SEM) for continuous variables with normal distribution. Continuous variables that did not follow a normal distribution curve were reported as median with 1st and 3rd quartile values (Q1, Q3). Categorical variables were reported as percentage (frequency) or percentage (numerator/denominator). The student’s t-test was used to compare continuous variables that followed a normal distribution. The Wilcoxon rank-sum test was used for variables that did not follow a normal distribution. The Pearson chi-square or Fisher’s exact tests were used to compare categorical variables. The Fisher’s exact test was used to generate more accurate p-values in cases when a cell contained a nonzero value ≤4, except for those variables that did not fit into a 2x2, 2x3, 2x4, or 3x3 table (patient race and tumor stage). In the tables, p-values that were generated using Fisher’s exact test are marked with an asterisk.

Cox regression analysis was used for survival analysis. Kaplan-Meier curves were generated to compare overall survival, and a log-rank test was used to compare overall survival time between patients in the Low BAR and High BAR groups. A receiver operator characteristics (ROC) curve was generated to determine the sensitivity and specificity of different BAR thresholds for predicting 30-day mortality. The area under the receiver operator characteristics curve (AUC) was calculated to evaluate the ability of higher BAR (as a continuous variable) to predict 30-day mortality. AUC represents the probability that a value can predict an outcome, and ranges in value from 0 to 1 with an AUC of 0 representing a model whose predictions are 100% incorrect and 1 representing a model whose predictions are 100% correct. An AUC>0.7 is generally accepted as an indicator that the parameter of interest is acceptable for predicting the outcome of interest. In our study, BAR was the value of interest, and 30-day mortality was the outcome of interest. Statistical analyses were carried out using the XLSTAT (Lumivero, Denver, CO, USA) and Real Statistics Resource Pack software, Release 7.6 (Real Statistics Using Excel, San Antonio, TX).

## Results

Out of 719 consecutive patients who underwent RAPL by one surgeon from September 2010 to March 2022, 319 patients were excluded from the study due to preoperative serum albumin levels not having been obtained prior to July 2013, resulting in a total of 400 study patients. The mean BUN in our cohort was 15.4 mg/dL, the mean albumin was 4.3 g/dL, and the mean BAR was 3.9 mg/g. The ROC curve revealed that the optimal BAR cutoff for predicting 30-day mortality was 6.25 mg/g, with a sensitivity of 23% and a specificity of 93%. The Low BAR group (BAR<6.25 mg/g) contained 369 patients, and the High BAR group (BAR ≥6.25 mg/g) contained 31/400 (7.8%) patients. The mean BUN was significantly higher in the High BAR group (p<0.0001), but the mean serum albumin was similar between groups (Table 1). The mean BAR in the Low BAR group was 3.6 mg/g, compared to 7.9 mg/g in the High BAR group (p<0.0001; Table 1).

**Table 1:** Preoperative Laboratory Values Data are presented as mean ± standard error of the mean (SEM). The p-values compare the Low BAR and High BAR groups.

Parameters	Total Cohort (N = 400)	Low BAR (N = 369)	High BAR (N = 31)	p-value
Blood Urea Nitrogen (BUN), mg/dL	15.4 ± 0.2	15.4 ± 0.2	33.3 ± 1.2	<0.0001
Serum Albumin, g/dL	4.30 ± 0.02	4.30 ± 0.02	4.24 ± 0.16	0.3036
BUN-to-Serum-Albumin Ratio (BAR), mg/g	3.92 ± 0.08	3.58 ± 0.05	7.90 ± 0.27	<0.0001

Patients characteristics

Demographics and preoperative comorbidities are compared in Table 2. Patients with High BAR were significantly older, had a higher proportion of males, and had higher incidences of hypertension, congestive heart failure, chronic anemia, diabetes mellitus, and chronic kidney disease compared to patients in the Low BAR group. The mean age in the High BAR group was 75.0 ± 1.3 years, compared to 69.0 ± 0.5 years in the Low BAR group (p=0.0009). The mean CCI scores were 1.35 ± 0.1 in the High BAR group and 0.76 ± 0.1 in the Low BAR group (p=0.0006).

**Table 2:** Patient Demographics and Preoperative Comorbidities Data are presented as mean ± standard error of the mean (SEM) or as % (frequency). Bolded p-values indicate statistical significance. *Fisher’s exact test was used to calculate p-value. BAR = blood-urea-nitrogen-to-serum-albumin ratio; BMI = body mass index; FEV1% = forced expiratory volume in one second as percentage of predicted; COPD = chronic obstructive pulmonary disease; MI = myocardial infarction; GERD = gastroesophageal reflux disease

Parameters	Low BAR (N=369)	High BAR (N=31)	p-value
Age, y	69.0 ± 0.5	75.0 ± 1.3	0.0009
Obese, BMI≥30kg/m^2^	28.7% (106)	22.6% (7)	0.4654
FEV1%	89.7 ± 1.0	88.5 ± 3.3	0.7436
Sex			
Male	41.5% (153)	61.3% (19)	0.0004
Female	58.5% (216)	38.7% (12)	-
Race			
White	92.7% (342)	87.1% (27)	0.0832
African American	1.4% (5)	6.5% (2)	-
Asian or Pacific Islander	1.9% (7)	6.5% (2)	-
Native American	3.3% (12)	0.0% (0)	-
Other/Multiracial	0.8% (3)	0.0% (0)	-
Smoking Status			
Current Smokers	16.0% (59)	6.5% (2)	0.0992^*^
Former Smokers	64.0% (236)	83.9% (26)	-
Nonsmokers	20.1% (74)	9.7% (3)	-
Pulmonary Comorbidities			
Obstructive Sleep Apnea	10.3% (38)	12.9% (4)	0.5522^*^
Asthma	7.9% (29)	3.2% (1)	0.4952^*^
COPD	23.8% (88)	25.8% (8)	0.8324
Pulmonary Fibrosis	1.9% (7)	6.5% (2)	0.1488^*^
Pneumonia	6.5% (24)	3.2% (1)	0.7087^*^
Cardiovascular Comorbidities			
Carotid Stenosis	4.9% (18)	0.0% (0)	0.3816^*^
Cerebrovascular Accident	4.6% (17)	6.5% (2)	0.6513^*^
Peripheral Vascular Disease	4.9% (18)	9.7% (3)	0.2163^*^
Hypertension	50.7% (187)	83.9% (26)	0.0004
Hyperlipidemia	43.1% (159)	58.1% (18)	0.1069
Atrial Fibrillation	7.6% (28)	16.1% (5)	0.0969
Other Arrhythmias	11.4% (42)	12.9% (4)	0.7697^*^
Congestive Heart Failure	1.4% (5)	9.7% (3)	0.0181^*^
Coronary Artery Disease/MI	16.0% (59)	29.0% (9)	0.0633
Other Comorbidities			
Previous Cancers	40.4% (149)	41.9% (13)	0.8654
Bleeding Disorders	2.7% (10)	0.0% (0)	1.0000^*^
Chronic Anemia	2.7% (10)	12.9% (4)	0.0171^*^
Chronic Kidney Disease	3.5% (13)	29.0% (9)	<0.0001
End-Stage Renal Disease	0.5% (2)	3.2% (1)	0.2154^*^
GERD	27.6% (102)	32.3% (10)	0.5825
Diabetes Mellitus	13.3% (49)	32.3% (10)	0.0043
Pancreatitis	1.4% (5)	6.5% (2)	0.0956^*^
Cirrhosis	0.8% (3)	0.0% (0)	1.0000^*^
Charlson Comorbidity Index	0.76 ± 0.1	1.35 ± 0.1	0.0006

Table 3 displays patients’ tumor size, primary pathology, tumor histology, tumor grade, nodal status, and pathologic stage. The majority of patients were primary lung cancer, stage IA, adenocarcinoma, and graded as well- or moderately differentiated, with a mean tumor size of 3.1 cm and pathologic nodal status of pN0. Of the patients with primary lung cancers, 69.2% in the Low BAR and 76.7% in the High BAR groups had pathologic nodal status of pN0, with pN2 being the second most common (16.5% in Low BAR vs. 23.3% in High BAR groups), and pN1 being the least common pathologic nodal status (14.2% in Low BAR vs. 0.0% in High BAR groups; p=0.0370; Table 3). There were no other significant differences in tumor characteristics between the BAR groups.

**Table 3:** Tumor Characteristics Data are presented as mean ± standard error of the mean (SEM) or % (numerator/denominator). BAR = blood-urea-nitrogen-to-serum-albumin ratio; AJCC v8 = American Joint Committee on Cancer, version 8

Parameters	Low BAR (N = 369)	High BAR (N = 31)	p-value
Tumor Size, cm	3.1 ± 0.1	3.5 ± 0.4	0.3266
Tumor Pathology			
Primary Lung Cancer	95.1% (351/369)	96.8% (30/31)	1.000^*^
Metastasis	3.8% (14/369)	3.2% (1/31)	-
Other Pathology	1.1% (4/369)	0.0% (0/31)	-
Lung Cancer Histology			
Adenocarcinoma	67.8% (238/351)	53.3% (16/30)	0.1204^*^
Squamous Cell Carcinoma	18.2% (64/351)	36.7% (11/30)	-
Neuroendocrine Carcinoma	10.0% (35/351)	6.7% (2/30)	-
Other Lung Cancer	4.0% (14/351)	3.3% (1/30)	-
Lung Cancer Grade			
Well-Differentiated	30.8% (108/351)	20.0% (6/30)	0.5572^*^
Moderately-Differentiated	43.9% (154/351)	53.3% (16/30)	-
Poorly-Differentiated	24.5% (86/351)	26.7% (8/30)	-
Undifferentiated	0.9% (3/351)	0.0% (0.30)	
Lung Cancer Pathologic Nodal (pN) Status			
pN0	69.2% (243/351)	76.7% (23/30)	0.0370^*^
pN1	14.2% (50/351)	0.0% (0/30)	-
pN2	16.5% (58/351)	23.3% (7/30)	-
Lung Cancer Pathologic Stage (AJCC v8)			
IA	43.0% (151/351)	30.0% (9/30)	0.1276
IB	12.8% (45/351)	23.3% (7/30)	-
IIA	6.0% (21/351)	133.3% (4/30)	-
IIB	16.8% (59/351)	10.0% (3/30)	-
IIIA	17.1% (60/351)	13.3% (4/30)	-
IIIB	4.3% (15/351)	10.0% (3/30)	-

The prognostic ability of High BAR

**Figure 1:**
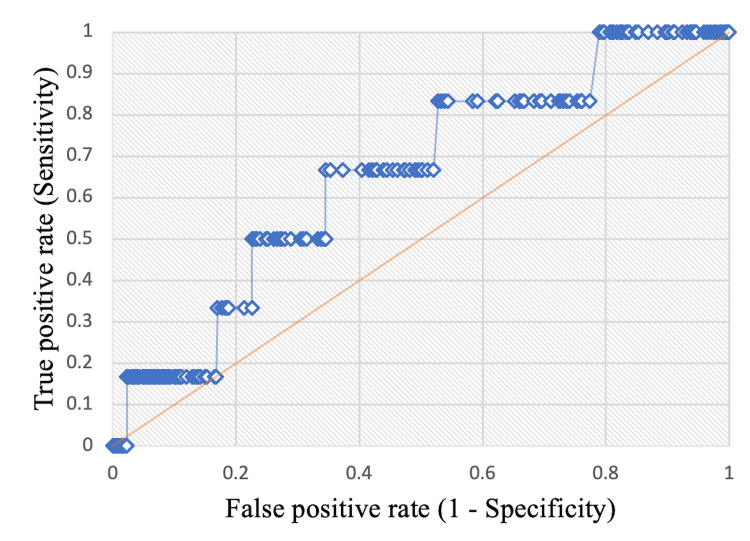
Receiver-operator-characteristics (ROC) curve with area-under-the-curve (AUC) analysis evaluating the ability of BAR to predict 30-day mortality. The ROC curve was generated to determine the sensitivity and specificity of different BAR thresholds for predicting 30-day mortality. The AUC was calculated to evaluate the ability of higher BAR to predict 30-day mortality. AUC represents the probability that a value can predict an outcome, and ranges in value from 0 to 1 with an AUC of 0 representing a model whose predictions are 100% incorrect and 1 representing a model whose predictions are 100% correct. An AUC>0.7 is generally accepted as an indicator that the parameter of interest is acceptable for predicting the outcome of interest. In our study, AUC=0.655 (95% confidence interval [CI]: 0.435-0.875; p=0.166).

The Low BAR and High BAR groups were similar with regard to the proportion of pulmonary and cardiovascular complications experienced postoperatively (Table 4). There were no differences in perioperative complications and outcomes, including discharge disposition (p=0.5690), in-hospital mortality (p=1.0000), and 30-day mortality (p=0.3912; Table 4). AUC analysis showed that higher preoperative BAR was not a predictor of 30-day mortality (AUC, 0.655, 95% CI, 0.435-0.875, p=0.166; Figure 1).

**Table 4:** Complications and Outcomes Data are presented as median [1st quartile, 3rd quartile] or as % (frequency). Bolded p-values indicate statistical significance. *Fisher’s exact test was used to calculate p-value; **Transferred to long-term acute care or rehabilitation facility, discharged to assisted-living facility or to hospice, or died. BAR = blood-urea-nitrogen-to-serum-albumin ratio

Parameters	Low BAR (N = 369)	High BAR (N = 31)	p value
Intraoperative Complications			
Any Intraoperative Complication	4.1% (15)	3.2% (1)	1.0000^*^
Bleeding (Pulmonary Artery)	2.2% (8)	0.0% (0)	1.0000^*^
Bleeding (Pulmonary Vein)	1.1% (4)	0.0% (0)	1.0000^*^
Bleeding (Other)	1.1% (4)	0.0% (0)	1.0000^*^
Tracheal/Bronchial Injury	0.5% (2)	3.2% (1)	0.2154^*^
Diaphragm Injury	0.0% (0)	0.0% (0)	-
Phrenic Nerve Injury	0.0% (0)	0.0% (0)	-
Recurrent Laryngeal Nerve Injury	0.0% (0)	0.0% (0)	-
Robotic-Associated Complication	3.5% (13)	3.2% (1)	1.0000^*^
Any Postoperative Complication	40.9% (151)	41.9% (13)	0.9439
Pulmonary Postoperative Complications			
Prolonged Air Leak for >5 Days	26.6% (98)	25.8% (8)	0.9274
Chyle Leak	6.5% (24)	0.0% (0)	0.2399^*^
Hemothorax	0.5% (2)	0.0% (0)	1.0000^*^
Effusion or Empyema	6.0% (22)	3.2% (1)	1.0000^*^
Pneumothorax after CT Removal	2.4% (9)	0.0% (0)	1.0000^*^
Hypoxia requiring Home O2 Supplement	1.9% (7)	6.5% (2)	0.1677^*^
Pulmonary Embolism	0.8% (3)	0.0% (0)	1.0000^*^
Aspiration	1.6% (6)	0.0% (0)	1.0000^*^
Pneumonia	5.1% (19)	0.0% (0)	0.3823^*^
Mucous Plug requiring Intervention	3.8% (14)	6.5% (2)	0.6309^*^
Respiratory Failure requiring Intervention	2.4% (9)	3.2% (1)	0.5725^*^
Cardiovascular Postoperative Complications			
Atrial Fibrillation	19.2% (71)	25.8% (8)	0.3778
True New-Onset Atrial Fibrillation	12.2% (45)	9.7% (3)	1.0000^*^
Shock/Multi-Organ System Failure	0.5% (2)	0.0% (0)	1.0000^*^
Cardiopulmonary Arrest	0.0% (0)	0.0% (0)	-
Myocardial Infarction	0.5% (2)	0.0% (0)	1.0000^*^
Cerebrovascular Accident	0.3% (1)	0.0% (0)	1.0000^*^
Hypotension	3.8% (14)	12.9% (4)	0.0598^*^
Perioperative Outcomes			
Estimated Blood Loss (EBL), mL	150 [94.0, 300.0]	150 [94.0, 213.0]	0.1431
Skin-to-Skin Operative Time, min	171 [141.0, 206.0]	177 [151.0, 217.0]	0.1087
Overall Conversions to Thoracotomy	3.5% (13)	3.2% (1)	1.0000^*^
Urgent Conversions to Thoracotomy	2.4% (9)	0.0% (0)	1.0000^*^
Chest Tube (CT) Duration, d	3.0 [2.0, 6.0]	4.0 [3.0, 6.0]	0.4540
Discharged with CT Valve	10.8% (40)	0.0% (0)	0.0583^*^
Hospital Length of Stay (LOS), d	4.0 [3.0, 6.0]	4.0 [3.0, 7.0]	0.5572
Unfavorable Disposition^**^	2.4% (9)	3.2% (1)	0.5690^*^
In-Hospital Mortality	1.1% (4)	0.0% (0)	1.0000^*^
30-Day Mortality	1.4% (5)	3.2% (1)	0.3912^*^

**Figure 2:**
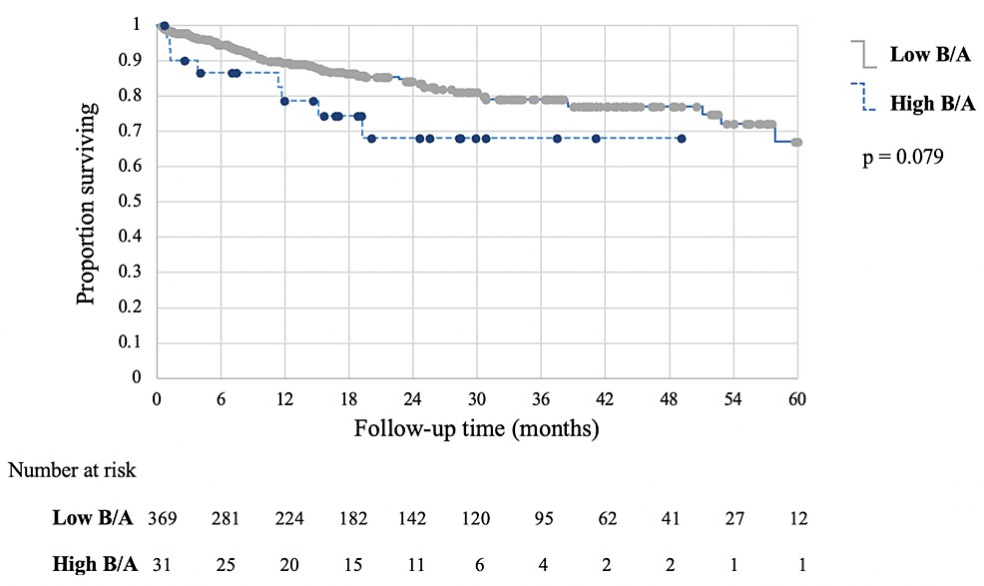
Kaplan-Meier Curves Showing Overall Survival by BAR Subgroup. Kaplan-Meier survival curves for patients with Low BAR <6.25 mg/g (solid line) versus High BAR ≥6.25 mg/g (dashed line). The five-year overall survival estimate is 78.0% in the High BAR group and 77.0% in the Low BAR group. The log-rank p-value comparing overall survival between the Low BAR and High BAR groups was p=0.079. Median overall survival times were not reached during the follow-up period. The numbers at risk noted below the x-axis at six-month intervals denote the number of patients still alive and who had subsequent follow-up (i.e., not yet censored). At five years postoperatively, 3.3% (12/369) of the Low BAR group and 3.2% (1/26) of the High BAR group remained at risk. BAR = blood-urea-nitrogen-to-serum-albumin ratio

Kaplan-Meier analysis showed that median overall survival times were not reached during the follow-up period. The five-year overall survival estimate is 78.0% in the High BAR group and 77.0% in the Low BAR group (Figure 2). Log-rank p-value comparing overall survival was 0.079.

## Discussion

Contrary to the majority of the literature, which reports worse outcomes among patients with elevated BAR, especially higher rates of in-hospital mortality and 30-day mortality, our data shows no difference in short- or long-term outcomes. This difference is likely due in part to the fact that most studies in the current literature included sicker patients compared to our cohort, with a higher average BAR, more comorbidities, and/or acutely worse laboratory values [4-16]. Many studies focused on critically ill patients, such as those admitted to the ICU with lung cancer, CHF, or acute pulmonary emboli [4,8,15].

Existing studies also often used AUC analysis to determine the optimal BAR value for predicting mortality in their cohort. Dundar et al. [14] studied BAR in older emergency department patients and found the same optimal cutoff of 6.25 mg/g as in our cohort. However, in their study, the AUC for BAR predicting in-hospital mortality was 0.793, which is an acceptable value showing reasonable prognostic power of BAR in their cohort [14]. Peng et al. studied BAR in intensive care unit patients with lung cancer and also found a reasonable AUC for BAR of 0.720 [15]. In our study, the specificity of the 6.25 mg/g BAR cutoff was excellent at 93%; however, the sensitivity was only 23%, indicating a possibly high number of false negatives. The low sensitivity, in combination with the less critically ill state of our patient cohort and the small number of overall deaths, likely contributed to our insignificant AUC value for BAR of 0.655. Only four patients in the Low BAR group and zero patients in the High BAR group passed away during their hospital stay. At 30 days postoperatively, only five patients in the Low BAR group and one patient in the High BAR group had passed away. Some existing studies also compared survivors to non-survivors, an analysis we were unable to reasonably do.

There were limitations to this study. The single-center, single-surgeon aspect of our study reduces the generalizability of our results. The study design was retrospective, which resulted in a cohort of 400 patients, reduced from 719 due to the missing preoperative serum albumin values for 319 patients. Regarding the original 719 patients, serum albumin levels were rarely available in the electronic medical record of patients undergoing surgery prior to 2015, then became consistently available after April 2015. In our cohort of 400 patients, 31 had a BAR ≥6.25 mg/g. The small number of patients in the High BAR group may have contributed to the statistically significant difference in the distribution of pathologic nodal status among the groups, as none of the patients with primary lung cancer in the High Bar group had pN1 nodal status. Similarly, the relatively small number of High BAR patients may have contributed to a lack of statistical significance in the outcome variables. The Low BAR group included 11.9 times the number of patients in the High BAR group. However, other researchers, such as Fang et al., analyzed BAR in a cohort with a similar imbalance and found significant results [8]. In the future, we aim to include more patients in our cohort.

## Conclusions

Lung cancer remains the number one cause of cancer death worldwide, and efforts to predict and optimize patient outcomes are warranted. Despite the physiologic importance of serum albumin and BUN, a preoperative BAR ≥6.25 mg/g did not predict worse outcomes after RAPL in lung cancer patients. Based on the present study, we would not be able to use the BAR to predict in-hospital or 30-day mortality in patients undergoing this procedure at our cancer center. Further study should be done to further confirm or contradict other reports that elevated BAR can be used as a prognostic tool in patients undergoing tumor resection, specifically those with lung cancer.
